# Abnormal coactivation of knee and ankle extensors is related to changes in heteronymous spinal pathways after stroke

**DOI:** 10.1186/1743-0003-8-41

**Published:** 2011-08-02

**Authors:** Joseph-Omer Dyer, Eric Maupas, Sibele de Andrade Melo, Daniel Bourbonnais, Robert Forget

**Affiliations:** 1 Centre de recherche interdisciplinaire en réadaptation du Montréal métropolitain, Institut de réadaptation Gingras-Lindsay de Montréal, 6300 avenue Darlington, Montréal, H3S 2J4, Canada; 2 École de réadaptation, Faculté de médecine, Université de Montréal, C.P. 6128, Succursale Centre-Ville, Montréal, H3C 3J7, Canada; 3 Centre Mutualiste de Rééducation Fonctionnelle, Laboratoire de Physiologie de la Posture et du Mouvement, Centre Universitaire JF Champollion, Place de Verdun, Albi, 81012, France; 4 Université Paul Sabatier, Toulouse III, Route de Narbonne, Toulouse, 31062, France

**Keywords:** Hemiparesis, Extension synergy, Sensory afferents, Isometric strength, Spinal Circuits, Propriospinal

## Abstract

**Background:**

Abnormal coactivation of leg extensors is often observed on the paretic side of stroke patients while they attempt to move. The mechanisms underlying this coactivation are not well understood. This study (1) compares the coactivation of leg extensors during static contractions in stroke and healthy individuals, and (2) assesses whether this coactivation is related to changes in intersegmental pathways between quadriceps and soleus (Sol) muscles after stroke.

**Methods:**

Thirteen stroke patients and ten healthy individuals participated in the study. Levels of coactivation of knee extensors and ankle extensors were measured in sitting position, during two tasks: maximal isometric voluntary contractions in knee extension and in plantarflexion. The early facilitation and later inhibition of soleus voluntary EMG evoked by femoral nerve stimulation were assessed in the paretic leg of stroke participants and in one leg of healthy participants.

**Results:**

Coactivation levels of ankle extensors (mean ± SEM: 56 ± 7% of Sol EMG max) and of knee extensors (52 ± 10% of vastus lateralis (VL) EMG max) during the knee extension and the ankle extension tasks respectively were significantly higher in the paretic leg of stroke participants than in healthy participants (26 ± 5% of Sol EMG max and 10 ± 3% of VL EMG max, respectively). Early heteronymous facilitation of Sol voluntary EMG in stroke participants (340 ± 62% of Sol unconditioned EMG) was significantly higher than in healthy participants (98 ± 34%). The later inhibition observed in all control participants was decreased in the paretic leg. Levels of coactivation of ankle extensors during the knee extension task were significantly correlated with both the increased facilitation (Pearson r = 0.59) and the reduced inhibition (r = 0.56) in the paretic leg. Measures of motor impairment were more consistently correlated with the levels of coactivation of biarticular muscles than those of monoarticular muscles.

**Conclusion:**

These results suggest that the heteronymous pathways linking quadriceps to soleus may participate in the abnormal coactivation of knee and ankle extensors on the paretic side of stroke patients. The motor impairment of the paretic leg is strongly associated with the abnormal coactivation of biarticular muscles.

## Background

Stroke patients often present a pathological extension synergy in the affected leg while attempting to move voluntarily [1]. This synergy is characterized by a stereotypical simultaneous activation of leg extensors, which is often referred as abnormal coactivation and may result in coupled movements of the hip, knee and ankle in extension during various tasks such as gait [1-4]. In the present paper, we will use the term "coactivation" to describe the simultaneous EMG activity in the knee and ankle extensor muscles and not the term "cocontraction". Since Sir Charles Sherrington formulated the principle of reciprocal inhibition, the latter has mostly been used to refer to the simultaneous activation of antagonist muscles.

As part of this extension synergy, the coactivation of knee and ankle extensors may have a major effect on function. Since these anti-gravity muscles have a normal out-of-phase activation during gait [5,6], their abnormal coactivation should be considered in the evaluation of walking disorders of the hemiparetic leg [7]. Although improper coactivation of leg extensors is clinically described in stroke and in various lesions of the central nervous system [1,8,9], only a few studies have quantified this coactivation.

In patients with central nervous system lesions, abnormal synergistic coactivations have been mostly measured during isolated contractions in static conditions [10-12]. In such tasks, where constraints due to movement are reduced and where the central nervous system has fewer parameters to control, the relationship between the coupling of the torques generated across the joints and the pattern of muscular recruitment is more easily interpreted than in dynamic tasks [11]. Some abnormal patterns of muscular recruitment and torque generation that are compatible with the pathological extension synergy have been shown in static conditions in cerebral palsy [11,13] and after stroke [14]. Regarding stroke, only a few studies have quantified abnormal coactivations at the paretic leg and no study has related them to motor impairments. Furthermore, the mechanisms underlying the abnormal synergistic activation of leg extensors in hemiparesis are still unclear.

Spinal interneurones are part of basic sensorimotor mechanisms that integrate descending and peripheral inputs. They can modulate the activity of motoneurones of muscles acting at the same joint or at different joints. Several studies have linked the malfunction of these pathways to motor deficits on the paretic side of stroke individuals. Modifications of the reciprocal inhibition of antagonist muscles acting at the same joint have been associated with changes in muscle tone [15], hyperreflexia [16] and the level of motor recovery in hemiparesis [17]. Heteronymous pathways are spinal pathways that can regulate the activity of motoneurones acting at different joints and thus, contrarily to homonymous pathways, link different spinal levels [18]. Changes in transmission in these propriospinal pathways are thought to contribute to incoordination of the paretic arm [19]. Alterations in such pathways have been documented at rest in the lower limb [20,21] and during gait [22] in stroke patients. In response to the stimulation of quadriceps muscles afferents, using femoral nerve stimulation, an abnormal increase in the early facilitation and a decrease in the later inhibition of both soleus Hoffmann reflex (H reflex) and voluntary EMG have been found in hemiparesis consecutive to stroke [23,24]. Moreover, incoordination of the paretic leg has been correlated to this increased heteronymous facilitation [23]. The question then arises as to whether the abnormal coactivation of knee and ankle extensors in the paretic leg is related to the malfunction of intersegmental pathways linking quadriceps to soleus.

This study aims (1) to compare the level of coactivation of knee and ankle extensors during maximal isometric voluntary contractions between stroke and healthy individuals; (2) to assess whether this coactivation is related to changes in the spinal pathways controlling heteronymous modulation of soleus activity by femoral nerve stimulation at the paretic leg. A preliminary report of the findings has been presented elsewhere [25].

## Methods

### Participants

Thirteen stroke patients with chronic hemiparesis (mean ± SD: 49 ± 15 years; 7 males, 6 females) and ten healthy individuals (44 ± 13 years; 8 males, 2 females) of similar age (p = 0.5) participated in the study. All participants gave their written informed consent to the study, which had been approved by the internal ethics committee of the institutions of the Center for interdisciplinary research in rehabilitation of greater Montreal. Stroke participants were recruited based on the following inclusion criteria: a single cerebrovascular accident involving the motor cortex, internal capsule or sub-cortical areas as documented by brain imagery and resulting in motor deficits of abrupt onset affecting the contralateral leg. All patients tested had detectable patellar and Achilles tendon reflexes in the paretic leg. Moreover, all participants were able to produce sustained voluntary contractions of knee and ankle extensors, in order to perform the experimental tasks, which consisted of pushing on a pad with the leg and pressing on a fixed platform with the forefoot, in sitting position. Individuals with stroke were excluded if they were on antispastic, anxiolytic or antidepressant medication at the time of the study, or if they had receptive aphasia, hemispatial neglect, or passive range of motion limitation of the paretic leg that could interfere with the experimental positioning. Moreover, participants with stimulators (e.g. pacemaker) or metallic implants were excluded, as were those with orthopaedic or neurological disorders other than stroke.

### Clinical assessment

Prior to the experimental sessions, stroke participants were evaluated for level of motor coordination, level of motor impairment, degree of spasticity at the paretic leg and self-selected comfortable gait speed. The level of motor coordination of the paretic lower limb was measured using the Lower Extremity Motor Coordination Test (LEMOCOT), validated for stroke individuals [26]. In this test, participants are seated and instructed to alternately touch with their foot, as fast and as accurately as possible, two standardized targets placed 30 cm apart on the floor, for a 20-second period. The LEMOCOT score was calculated as the number of times the subject touched the two targets. The level of motor impairment was measured using the reliable Chedoke-McMaster Stroke Assessment (CMSA) subscale for motor recovery stage at the paretic foot [27]. This subscale ranges from 1 (no residual motor function) to 7 (no residual motor impairment) and is based on Brunnstrom's stages of motor recovery of the lower extremity [1]. The degree of spasticity of the paretic ankle was measured with a reliable composite spasticity index (CSI) designed for stroke patients. Practical considerations in the use of CSI are described by Levin and Hui-Chan (1993) [28]. Briefly, this index is a 16-point scale that includes subscales measuring the amplitude of the Achilles' tendon tap jerk (4-point), duration of the clonus (4-point) and the resistance to passive stretching of ankle extensors at moderate speed (8-point). Interval values of 1-5, 6-9, 10-12 and 13-16 correspond to absent, mild, moderate and severe spasticity, respectively [28,29]. The self-selected overground comfortable walking speed was evaluated using a stopwatch to measure the time taken to cover a 10-meter distance (mean of three trials) without technical assistance (cane, walker) or orthosis [30,31]. Table 1 presents demographic data and clinical characteristics of stroke participants with heterogeneous levels of disability. This clinical evaluation was followed by the experimental session, which comprised two assessments performed in random order, the same day: 1) evaluation of the coactivation of knee and ankle extensors on a dynamometer and 2) electrophysiological exploration of the heteronymous modulation.

**Table 1:** Demographic and clinical data for participants with stroke

Participant	Age/gender	Side of brain lesion	Time since stroke (months)	CMSA at Foot (/7)	LEMOCOT (no. of hits on targets)	CSI (/16)	Gait speed (m/s)
1	57/M	L	79	5	31	10	0.7
2	24/M	L	97	3	5	13	0.3
3	43/F	R	38	6	26	6	1.1
4	59/M	L	76	7	23	5	0.7
5	45/M	R	79	7	20	8	1.3
6	72/M	L	48	5	19	6	0.9
7	59/F	L	57	7	19	7	0.6
8	43/F	R	90	3	19	8	1.0
9	72/M	L	96	4	13	7	0.6
10	28/F	R	108	4	12	12	0.9
11	45/F	L	96	7	52	5	1.2
12	54/M	R	149	2	1	7	0.5
13	30/F	R	103	5	10	11	1.1

**LEMOCOT: **Lower Extremity Motor Coordination Test; **CMSA at Foot**: Chedoke-McMaster Stroke Assessment at the foot; **CSI: **Composite Spasticity Index

### Assessment of the coactivation

#### Experimental set-up and instrumentation

**Figure 1:**
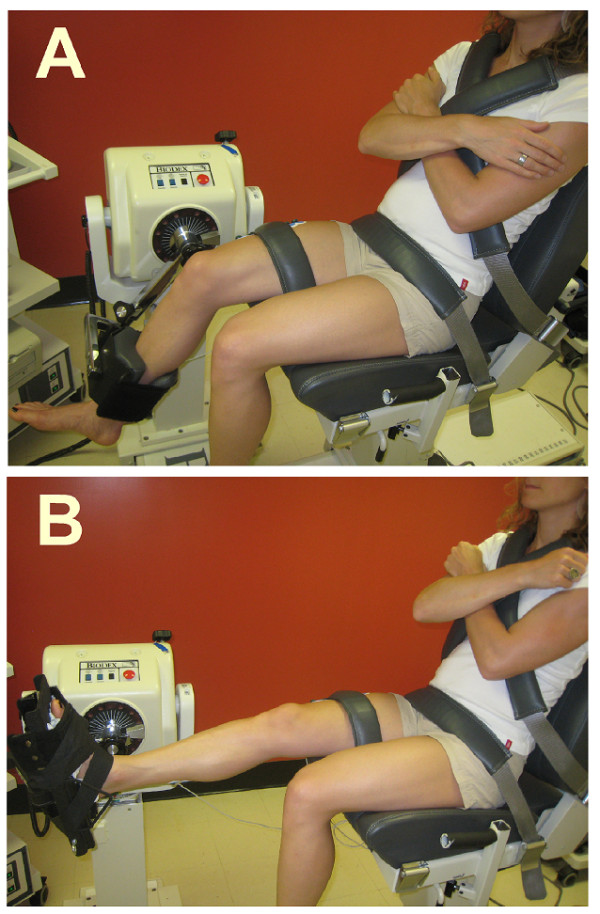
**Photographs of the experimental set-up for the assessment of the coactivation**. Participants were comfortably secured in sitting position with the chest, pelvis and the tested leg being firmly anchored to the Biodex chair. A. For the knee extension task, the input axis of the dynamometer was adjusted to align with the knee axis of rotation through the lateral femoral condyle and the foot was left free. B. For the plantarflexion task, the ankle joint (lateral malleolus) was aligned with the input axis of the dynamometer and fixed in plantarflexion (110°) using the standard Biodex ankle unit attachment. EMG activity was recorded from vastus lateralis (VL), rectus femoris (RF), soleus (Sol) and gastrocnemius lateralis (GL).

A Biodex system 3 dynamometer (Biodex Medical Systems, Inc., Shirley, New York) was used to measure the torques generated during two tasks: the maximal isometric knee extension and plantarflexion. Prior to each session, the dynamometer was calibrated. Figure 1 presents the experimental set-up. Participants were comfortably seated on the Biodex accessory chair. The paretic leg was tested in stroke participants. The tested leg was randomized in control participants since previous pilot experiments showed no difference in the level of coactivation of knee and ankle extensors between the dominant and non-dominant sides of healthy subjects during maximal isometric knee extension and ankle extension tasks. For the knee extension task, the knee was flexed at 60°, which has been demonstrated to be the angle of maximal isometric knee extension force generation [32,33]. Torque levels generated were automatically adjusted for gravity by the Biodex software. For the plantarflexion task, all participants were seated with the knee slightly flexed (10°) and the ankle was fixed in plantarflexion (110°) [34].

EMG activities of soleus (Sol), lateral gastrocnemius (GL), rectus femoris (RF) and vastus lateralis (VL) were simultaneously recorded. The skin was carefully prepared before placement of the disposable, self-adhesive, Ag/Ag-Cl surface electrodes (Ambu ^® ^Blue Sensor M) fixed in a bipolar configuration (2-cm center-to-center interelectrode distance leaving 8 mm spacing between the recording areas) over the belly of each recorded muscle. EMG signals were tested for crosstalk by performing standard muscle testing, rapid alternating movements and using the minimal interelectrode distance. EMG activities were collected using a telemetric system (Telemyo 900, NORAXON Telemyo System, Scottsdale, AZ), relayed to a battery powered amplifier (2000x) with a bandwidth of 10 to 500 Hz and transmitted to a receiver interfaced with a PC card. These signals were acquired at a sampling rate of 1200 Hz using software constructed on a LabVIEW 5.0 platform (National Instruments) and stored on computer for later analysis.

#### Experimental protocol for coactivation assessment

Prior to each task and to any data collection, all participants had a 5-minute practice during which they produced submaximal isometric contractions (4-7 trials) on the dynamometer. After the practice, a 5-minute rest period ensued. Before performing each experimental task, participants were first instructed to relax completely (background EMG below 5 μV). During the assessment, they were instructed to fold their arms across their chest and were verbally encouraged to reach their maximal isometric voluntary contraction as soon as possible after a "GO" signal and to hold a steady contraction for 4 s, with the visual feedback of the ongoing torque generated [35,36]. A minimum 2-minute rest period was given after each trial. For each task, the EMG signals and the torque output were simultaneously recorded for 10 s after the GO signal for three trials.

#### Data analysis for coactivation assessment

**Figure 2:**
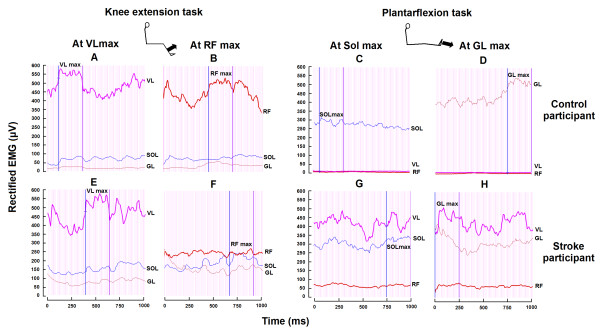
**Levels of EMG activity of knee and ankle extensors during the knee extension and plantarflexion tasks in a healthy and a stroke participant**. Traces of rectified EMG activities of vastus lateralis (VL), rectus femoris (RF), soleus (Sol) and gastrocnemius lateralis (GL) (expressed in μV) are presented on the paretic side of a stroke participant (lower row) and on the right side of a control participant (upper row). For each participant, traces are plotted for one second of contraction during maximal knee extension (left panel) and during maximal plantarflexion (right panel). Vertical bars represent the 250-ms time windows when maximal EMG activity was achieved in VL and RF during the knee extension task and in Sol and GL during the plantarflexion task.

All analyses were performed off-line. EMG signals were filtered using a zero-phase shift fourth-order digital Butterworth band-pass filter (20-125 Hz) and were full-wave rectified to obtain smoothed linear envelopes. The maximal torques in knee extension and plantarflexion were determined by averaging the maximal torque outputs of three trials for each task. Figure 2 presents examples of EMG traces during the two tasks in a control and a stroke participant. For the knee extension task, the coactivation levels of the plantarflexors with reference to the quadriceps were determined, for each trial, by the mean EMG activity of soleus and gastrocnemius lateralis within the 250-ms time windows when maximal mean EMG activity was reached at VL (Sol at VL_max_; GL at VL_max_) and at RF (Sol at RF_max_; GL at RF_max_). For the plantarflexion task, the coactivation levels of the quadriceps with reference to the plantarflexors were determined, for each trial, by the mean EMG activity of VL and RF within the 250-ms time windows when maximal mean EMG activity was reached at Sol (VL at Sol_max_; RF at Sol_max_) and at GL (VL at GL_max_; RF at GL_max_). For each muscle, the mean coactivation level was the average of the three trials per task and expressed as a percentage of the maximal EMG achieved in that muscle across the three trials of knee extension for VL and RF, and of plantarflexion for Sol and GL.

### Electrophysiological evaluation of the heteronymous modulation

#### Experimental set-up and instrumentation

Participants were comfortably seated in a position similar to the one during the plantarflexion task of the assessment of coactivation in an adjustable reclining armchair with the foot strapped with Velcro to a fixed pedal. The femoral nerve was stimulated with a 1-ms duration monophasic rectangular pulse (Grass S88 stimulator) delivered by a cathode (half-ball of 2-cm diameter) at the femoral triangle and an anode (11.5 cm × 8 cm) placed at the postero-lateral aspect of the buttock. Stimulation intensity was progressively increased to determine the thresholds of the H reflex and of the M response (MT) for vastus lateralis. The intensity was then maintained at 2 × MT of vastus lateralis for the rest of the experiment. EMG activities of soleus and vastus lateralis were recorded (Grass, model 12 acquisition system) using bipolar surface electrodes (Beckmann, Ag-AgCl; 9 mm diameter) placed 2 cm apart (center-to-center). The recording electrodes were secured over the belly of vastus lateralis and soleus. EMG signals were first amplified (5000 x), then filtered (30-1000 Hz) (Grass, model 12 A 5) and finally, acquired at a sampling rate of 5 kHz. EMG signals were displayed on an oscilloscope and stored on computer for off-line analysis.

#### Experimental protocol for evaluation of the modulation

Participants were instructed to press with the forefoot on the fixed platform in order to produce isometric plantarflexions. The level of EMG activity of soleus during maximal isometric voluntary contractions in plantarflexion (EMG_max_) of 5-second duration was first determined for each participant (mean of three trials). All participants then produced isometric steady plantarflexions to activate soleus at 30% of EMG_max_. Throughout the experiment, an analogue voltmeter facing the participant displayed visual feedback of the level of voluntary activity achieved at soleus (rectified and integrated EMG activity surface) for baseline control. Contractions had to be maintained for at least 3 s and a minimum rest period of 20 s was allowed between each of them. Random stimulations of femoral nerve at 2 × MT of vastus lateralis were performed during these contractions so that stimulation occurred in about one out of three contractions. The interval between the onset of soleus activation and the stimulation was also randomized. This ensured that participants would not be able to predict at which contractions the stimulation would be applied, or exactly when it would occur after the onset of soleus activation. For each leg tested, unconditioned and conditioned voluntary EMG activities of ten trials of femoral nerve stimulation were recorded during soleus voluntary contractions.

#### Data analysis for evaluation of the modulation

Assessments of the heteronymous modulation were performed off-line. For each trial, soleus EMG was full-wave rectified for 100 ms before to 80 ms after the stimulation of the femoral nerve. The latency of the changes in soleus EMG was expressed in terms of the zero central delay, that is when the fastest femoral nerve Ia volley is expected to arrive at the segmental level of soleus motoneurone pool. This zero central delay was calculated for each participant from the latency of soleus H reflex and from the difference in afferent conduction time between homonymous and heteronymous Ia pathways [37,38]. The mean zero central delay of control participants (mean ± SEM: 24 ± 0.6 ms; ranging from 22 to 27 ms) was not different from that of stroke participants (23 ± 0.5 ms; ranging from 21 to 26 ms).

For both healthy and stroke participants tested in this study, early facilitation was found to peak within 6 ms after the zero central delay and to reach a maximal duration of 12 ms in healthy participants and 36 ms in some severely affected stroke participants. In healthy participants, the later inhibition could be observed as early as 6 ms after the zero central delay and lasted about 40 ms. Thus, in each participant, the level of facilitation was measured by the surface of soleus EMG within the window of analysis from 0 to 6 ms after the zero central delay (about 25 to 31 ms after femoral nerve stimulation). The later inhibition was assessed within 3 consecutive time windows of analysis of 12-ms duration each, from 12 to 24 ms, 24 to 36 ms and 36 to 48 ms after the zero central delay (about 37 to 73 ms after femoral nerve stimulation). Facilitation and inhibition levels were measured for each trial, at each time window, as the difference between the integrated rectified EMG after the conditioning stimulation (conditioned EMG) and before the stimulation (unconditioned EMG). This difference was expressed as a percentage of the control EMG measured within a window of 100 ms-duration just before the stimulation and then normalized for the duration of the time windows of analysis. Mean facilitation and inhibition of soleus voluntary EMG were assessed on ten trials of isometric contraction.

### Statistical analysis

For the coactivation assessment, Mann-Whitney U-tests were used to compare the levels of coactivation between the two groups (stroke vs. healthy). Wilcoxon signed rank tests were performed to compare the levels of coactivation of different muscles within the same group. Spearman rank correlations were used to correlate the scores obtained for clinical tests of coordination (LEMOCOT), motor recovery (CMSA), spasticity (CSI) and gait speed with the coactivation levels measured in stroke individuals. For the electrophysiological evaluation, analysis of variance (ANOVA) using Scheffe's method were performed in order to determine whether there was significant facilitation and inhibition throughout the windows of analysis before and after femoral nerve stimulation. Mann-Whitney U-tests were used to compare the levels of modulation between the two groups. Wilcoxon signed rank tests were performed to compare the levels of soleus EMG before and after femoral nerve stimulation within group or participant. Pearson correlations were used to correlate the levels of coactivation at the paretic leg with the levels of heteronymous modulation. The Spearman rank test was performed to assess the correlations between clinical scores (LEMOCOT, CMSA and CSI) and electrophysiological data. P values ≤ 0.05 were considered significant. All statistical analyses were performed using the Statistical Package for Social Science (SPSS) software, version 17 for Windows.

## Results

### Coactivation of knee and ankle extensors

In both tasks tested, increased levels of coactivation were found in stroke participants compared to control participants. Figure 2 presents the EMG activity of knee and ankle extensors (rectified and expressed in μV) during one trial of the knee extension task and of the plantarflexion task, in a control participant and a stroke participant (#5 in Table 1). For the knee extension task, the EMG activity of Sol (expressed as a % of its maximal EMG) during maximal activations of VL and RF reached 29% (Figure 2A) and 27% (Figure 2B) respectively in the control participant and 49% (Figure 2E) and 69% (Figure 2F) in the stroke participant. For the plantarflexion task, the EMG activity of VL during maximal activations of Sol and GL reached 2% (Figure 2C) and 3% (Figure 2D) respectively in the control participant and 77% (Figure 2G) and 81% (Figure 2H) in the stroke participant.

**Figure 3:**
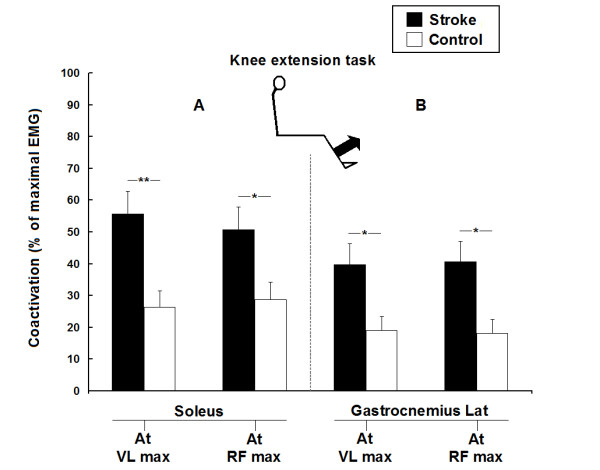
**Group comparisons of the coactivation of ankle extensors during the knee extension task in 13 stroke and 10 healthy participants**. Mean levels of coactivation of soleus (A) and gastrocnemius lateralis (B) (expressed as a % of their maximal EMG) during the maximal activation of vastus lateralis (at VL max) and rectus femoris (at RF max) for the knee extension task. Vertical bars = 1 SEM. Asterisks represent significant differences between the stroke group (black bars) and the control group (white bars) (* p ≤ 0.05; ** p ≤ 0.01).

Figure 3 shows the mean levels of coactivation of Sol and GL (expressed as a % of their maximal EMG) during maximal activations of VL and of RF, for the knee extension task in all participants. Coactivations of Sol observed in the stroke group were higher (P < 0.05) than in the control group (A). In one stroke participant with severely impaired coordination (#2 in Table 1), coactivation of Sol reached 104% during the knee extension task and was thus similar to the maximal voluntary activation of Sol during the plantarflexion task. Coactivations of GL observed in the stroke group were also higher (P < 0.05) than in the control group (B). Moreover, when levels of coactivation were compared between monoarticular and biarticular muscles, the mean level of coactivation of Sol (i.e. monoarticular) was higher than the coactivation of GL (i.e. biarticular) during maximal activation of quadriceps in both groups (P < 0.05). Maximal torque generated during the knee extension task in the stroke group (mean ± SD; 111 ± 33 Nm) was lower (P < 0.001) than in the control group (192 ± 54 Nm).

**Figure 4:**
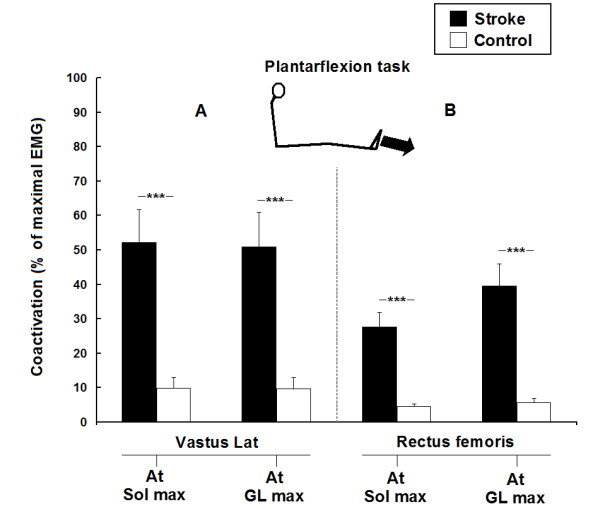
**Group comparisons of the coactivation of knee extensors during the plantarflexion task in 13 stroke and 10 healthy participants**. Mean levels of coactivation of vastus lateralis (A) and rectus femoris (B) (expressed as a % of their maximal EMG) during the maximal activation of soleus (at Sol max) and gastrocnemius lateralis (at GL max) for the plantarflexion task. Vertical bars = 1 SEM. Asterisks represent significant differences between the stroke group (black bars) and the control group (white bars) (* p ≤ 0.05; ** p ≤ 0.01).

Figure 4 shows mean levels of EMG coactivation of VL and RF (expressed as a % of their maximal EMG) during maximal activations of Sol and GL, for the plantarflexion task in all participants. Coactivations of both VL (A) and RF (B) in the stroke group were higher (P < 0.001) than in the control group. In one stroke participant with severely impaired coordination (#12 in Table 1), coactivation of VL reached 135% during the plantarflexion task and was thus higher than the maximal voluntary activation of VL during the knee extension task. Moreover, when levels of coactivation were compared between monoarticular and biarticular muscles, the mean level of coactivation of VL (i.e. monoarticular) was higher than the coactivation of RF (i.e. biarticular) during maximal activation of Sol in both groups (P < 0.05). Maximal torque generated during the plantarflexion task in the stroke group (mean ± SD; 64 ± 26 Nm) was lower (p < 0.001) than in the control group (125 ± 22 Nm).

### Heteronymous modulation across participants

**Figure 5:**
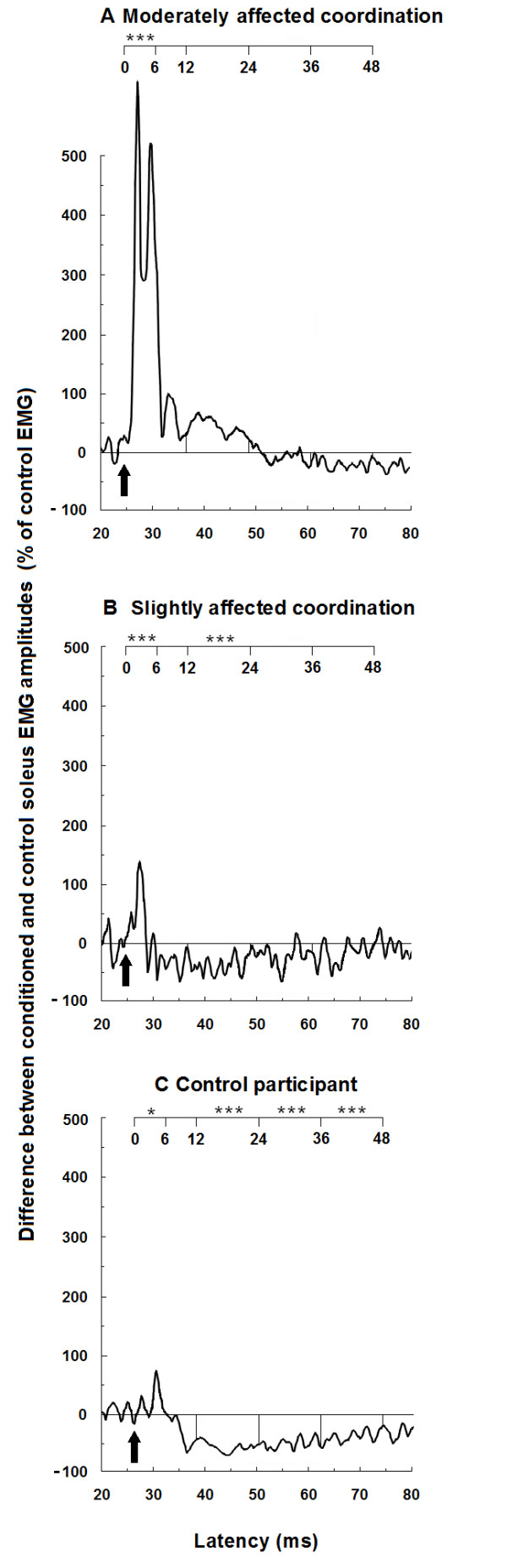
**Effects of femoral nerve stimulation on soleus voluntary activity in two stroke participants and a control participant**. Traces of averaged rectified soleus EMG activities of ten trials are presented on the paretic sides of stroke participants with moderately (A) and slightly (B) impaired coordination and on the right leg of a control participant (C). For each participant, traces are plotted against the latency presented from 20 ms to 80 ms after femoral nerve stimulation (lower scale), and from 0 to 48 ms after the zero central delay (upper scale). Arrows indicate the zero central delay, which is the expected time of arrival of the fastest femoral nerve Ia volley at the motoneurone level of soleus. Horizontal lines represent the mean amplitude of the unconditioned EMG activity before femoral nerve stimulation (baseline EMG level). The early facilitation was assessed within the time window from 0 to 6 ms after zero central delay. Asterisks represent significant modulations of soleus voluntary EMG within the four time windows of analysis from 0 to 6 ms, 12 to 24 ms, 24 to 36 ms and 36 to 48 ms after the zero central delay (* p ≤ 0.05; ** p ≤ 0.01; *** p ≤ 0.001).

An increase in the early facilitation and a decrease in the later inhibition of soleus voluntary EMG induced by femoral nerve stimulation was observed in stroke participants. Figure 5 shows the heteronymous modulation in stroke participants with moderately affected (# 3 in Table 1) and slightly affected coordination (# 11 in Table 1) and in a control participant. Within the time window from 0 to 6 ms after zero central delay, the facilitation observed in the moderately impaired stroke participant (mean ± SEM; 327 ± 66% of Sol unconditioned EMG surface) was higher (P < 0.05) than the facilitation in the slightly affected participant (155 ± 17%) and than in the control participant (117 ± 35%). In the next time window from 12 to 24 ms, the facilitative modulation observed in the moderately impaired participant (increase of 40 ± 21% of Sol EMG) was different (P < 0.05) from the inhibitions observed in the slightly impaired individual (decrease of 45 ± 7%) and in the control participant (decrease of 60 ± 7%). Within the next two time windows from 24 to 36 ms and from 36 to 48 ms, the inhibitions in the moderately affected stroke participant (decrease of 3 ± 9% and 18 ± 8% of Sol EMG, respectively) were lower (P < 0.05) than the inhibitions in the slightly affected stroke participant (32 ± 20% and 28 ± 29%, respectively) and than in the control participant (52 ± 8% and 37 ± 7%, respectively). Across all of the participants, a significant facilitation (Scheffe's method; P < 0.05) was observed from 0 to 6 ms after zero central delay in four out of ten control participants and in nine out of thirteen of those with stroke. Within the next three time windows, a significant heteronymous inhibition was observed in all of the control participants but was absent in four out of thirteen of those with stroke.

### Heteronymous modulation across groups

**Figure 6:**
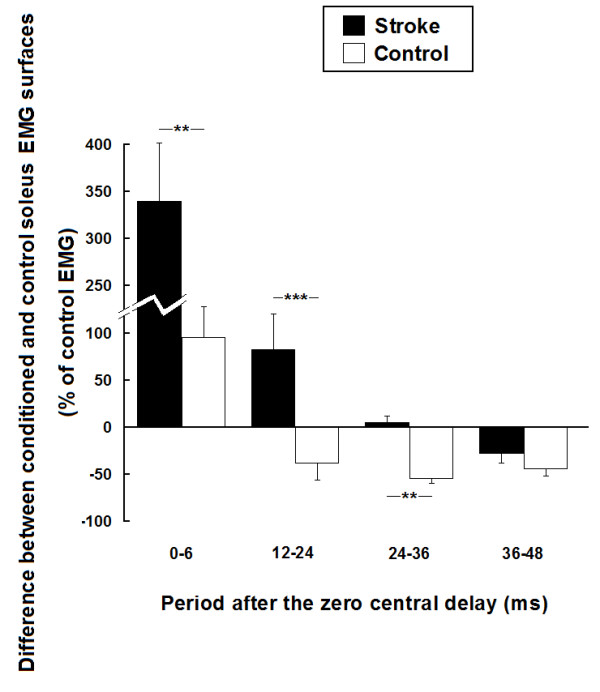
**Group comparisons of the effects of femoral nerve stimulation on soleus voluntary EMG activity in 13 stroke and 10 healthy participants**. Mean modulations of soleus voluntary EMG activity induced by femoral nerve stimulation for the stroke group (black bars) and the control group (white bars) (expressed as a % of soleus unconditioned EMG surface). Modulations are presented within the four time windows of analysis from 0 to 6 ms, 12 to 24 ms, 24 to 36 ms and 36 to 48 ms after the zero central delay. Positive values (i.e. above zero on the ordinate) denote facilitation and negative values denote inhibition. Vertical bars = 1 SEM. Asterisks represent significant difference in modulation between the control and the stroke participants (* p ≤ 0.05; ** p ≤ 0.01; *** p ≤ 0.001).

Figure 6 shows the mean heteronymous modulation observed in the stroke and control groups. Within the time window from 0 to 6 ms after the zero central delay, the early facilitation observed in the stroke group (mean ± SEM; 340 ± 62%) was greater (P < 0.01) than in the control group (98 ± 34%). The modulations observed within the next two time windows in the stroke group (facilitation of 82 ± 38% and 5 ± 13% of Sol EMG, respectively) were different (P < 0.01) from the inhibitions observed in the control group (decrease of 41 ± 15% and 55 ± 5% of Sol EMG, respectively). Within the last time window, the inhibition observed in the stroke group (decrease of 27 ± 10%) was not significantly different from the inhibition observed in the control group (decrease of 46 ± 8%).

### Correlations between coactivations and heteronymous modulations

Table 2 presents for both groups, the correlations between the coactivations observed during both maximal isometric voluntary contraction tasks and the heteronymous modulation of soleus within the first two time windows of analysis. In stroke participants only, the coactivation levels of Sol and GL at VL_max _during the knee extension task were both correlated with the modulations within the first two time windows of analysis. No correlations were found between coactivation levels during the plantarflexion task and levels of heteronymous modulation in either stroke or control participants. Moreover, no correlations were found between coactivation levels and the last two time windows of analysis of the heteronymous modulation (24-36 ms and 36-48 ms).

**Table 2:** Correlation coefficients (Pearson) between heteronymous modulations of Sol produced by femoral nerve stimulation and levels of coactivation in static tasks

Group	Modulations	Levels of coactivation							
	**Time windows** **(ms after ZCD)**	**Knee extension task**				**Plantarflexion task**			
		**Sol**		**GL**		**VL**		**RF**	
		**at VL_max_**	**at RF_max_**	**at VL_max_**	**at RF_max_**	**at Sol_max_**	**at GL_max_**	**at Sol_max_**	**at GL_max_**

**Stroke**	**(0-6)**	0.59*	0.46	0.59*	0.52	-0.07	-0.05	0.19	0.22
	**(12-24)**	0.56*	0.48	0.55*	0.41	0.15	0.16	0.30	0.35
									
**Control**	**(0-6)**	-0.28	-0.38	-0.17	-0.03	0.08	0.05	-0.06	-0.11
	**(12-24)**	-0.05	-0.16	-0.04	-0.02	-0.18	-0.19	-0.14	-0.16

**Sol: **Soleus; **GL: **Gastrocnemius Lateralis; **VL: **Vastus Lateralis; **RF: **Rectus Femoris; **ZCD: **Zero Central Delay; Significant correlations are in bold characters; *p ≤ 0.05

### Correlations with clinical measures

The level of coordination of the paretic leg (LEMOCOT) was correlated with comfortable gait speed (r = 0.56; P = 0.048) and level of motor recovery (CMSA) (r = 0.73; P = 0.005), and tended to correlate with degree of spasticity (CSI) (r = -0.55; P = 0.052). Table 3 shows the correlations between clinical scores and coactivation levels in stroke participants. For the knee extension task, GL coactivations were inversely correlated with coordination score (LEMOCOT), level of motor recovery (CMSA) and self-selected comfortable gait speed, and positively correlated with degree of spasticity (CSI). Sol coactivations were not correlated with any of the clinical measures, except for the coactivation of Sol at RF_max_, which was inversely correlated with comfortable gait speed. For the plantarflexion task, the amounts of coactivation of VL and RF were all inversely correlated with the coordination score. However, only the coactivations of RF were significantly correlated with level of motor recovery and degree of spasticity.

**Table 3:** Correlation coefficients (Spearman) between coactivation levels of knee and ankle extensors and clinical measures in stroke participants

Clinical tests	Levels of coactivation							
	**Knee extension task**				**Plantarflexion task**			
	**Sol**		**GL**		**VL**		**RF**	
	**at VL_max_**	**at RF_max_**	**at VL_max_**	**at RF_max_**	**at Sol_max_**	**at GL_max_**	**at Sol_max_**	**at GL_max_**

**LEMOCOT**	-0.15	-0.20	-0.76**	-0.75**	-0.58*	-0.55*	-0.72**	-0.55*
**CMSA_Foot_**	-0.33	-0.19	-0.75**	-0.73**	-0.42	-0.38	-0.66*	-0.69**
**CSI**	0.54	0.52	0.75**	0.68*	0.45	0.43	0.73**	0.87**
**Gait _speed_**	-0.47	-0.56*	-0.64*	-0.70**	-0.21	-0.20	-0.40	-0.43

**Sol **: Soleus; **GL**: Gastrocnemius Lateralis; **VL**: Vastus Lateralis; **RF**: Rectus Femoris; **LEMOCOT**: Lower Extremity Motor Coordination Test; **CMSA: **Chedoke-McMaster Stroke Assessment at the foot; **CSI: **Composite Spasticity Index; **Gait_Speed _: **Comfortable walking speed; Significant correlations are in bold characters; *p ≤ 0.05;** p ≤ 0.01

## Discussion

### Coactivation of leg extensors after stroke

The present study shows, in stroke individuals, an increased coactivation of knee and ankle extensors of the paretic leg during ankle and knee extensions, respectively. Only a few studies have quantified abnormal coactivations of leg extensors in stroke patients. An abnormal increased coactivation of knee and hip extensors has been found in standing position during maximal isometric extensions of the paretic knee and hip [39]. Stroke patients also demonstrated abnormal torques coupling between hip adduction and knee extension during submaximal isometric contractions while standing with the leg positioned as the toe-off position of gait [40].

One should point out that the effects of crosstalk could have influenced the levels of coactivation observed in the present study. However, these effects should be similar in stroke and control participants and thus cannot explain the difference in coactivation between the two groups. Moreover, we compared activity in a proximal (vastus lateralis or rectus femoris) muscle versus a distal (soleus or gastrocnemius) limb muscle considered to be relatively far apart and recorded with the smallest interelectrode distance possible. This makes crosstalk less probable than comparing muscles that are close together in the same limb segment such as antagonist muscles.

Our results showed coactivation levels of gastrocnemius lateralis in the paretic leg that were twice the levels found in healthy control subjects during knee extension. Similarly, coactivations in vastus lateralis were five times the levels found in control subjects during plantarflexion. These results concur with another study in which abnormal coactivations of gastrocnemius and rectus femoris were found during maximal knee extension and ankle extension after stroke, but in standing position [14].

Our study also points out that, in some stroke individuals with severely impaired coordination, the activation of a muscle can be greater during its synergistic coactivation with other muscles than during its activation while attempting to produce maximal torque in a specific direction at which that muscle is normally preferentially activated. One should consider that the present study did not assess the ability of participants to produce isolated muscle activation. Participants were allowed to choose whatever strategy they want, including to produce coactivations, in order to perform the tasks. This allows us to assess which muscles would be preferentially recruited during these tasks. It should also be noted that the positioning and support provided during the two tasks could have favoured abnormal muscle recruitments and coactivations in both groups. Our results correspond with those of Neckel et al., (2006) showing that, for leg extension in stroke subjects, the secondary torque generated during the maximal voluntary contraction at another joint can be greater than the maximal voluntary torque generated at that joint. This concept of a greater recruitment of a muscle group during its synergistic coactivation than while attempting to specifically recruit it has been used in clinical rehabilitation in the Brunnstrom and proprioceptive neuromuscular facilitation approaches of stroke [41,42].

The mechanisms underlying these abnormal coactivations are not well understood. Weakness, changes in supraspinal influences and dysfunction of spinal pathways have been suggested as possible mechanisms contributing to the development of synergistic coactivations after stroke [10]. There is no consensus on the relation between coactivations and weakness. On the one hand, it has been proposed that coactivations are adaptive compensations of the paretic limb, in which there is an unequal distribution of weakness across joints and muscles [10]. On the other hand, some evidence suggests that coactivations may contribute to weakness rather than be caused by a lack of strength of the paretic leg [39,43]. It has been shown that increased cocontractions of antagonists during ankle flexion and ankle extension contribute to the joint torque deficits in those directions in the paretic leg [14]. In the present study, cocontractions of agonist-antagonist pairs may have reduced the net maximal torques produced at the paretic leg and thus could have contributed to the weakness found in stroke participants. Changes in supraspinal influences after stroke can affect the ability to selectively activate muscles and thus may contribute to abnormal patterns of muscle activation. It has been suggested that an enlargement of the cortical areas activated during voluntary tasks may participate in the abnormal synergistic recruitment [10,44,45]. Moreover, our results point out that changes in the propriospinal circuits could affect these coactivations.

### Changes in heteronymous modulation after stroke

An increase in early heteronymous facilitation and a decrease in later inhibition of soleus voluntary EMG after femoral nerve stimulation were observed in the paretic leg of stroke individuals. Early heteronymous facilitation and later inhibition are thought to be mediated by intersegmental group Ia afferent excitation and recurrent inhibition from femoral nerve to soleus motoneurones, respectively [37,38,46-48]. Several spinal mechanisms may contribute to modifications in heteronymous modulation after stroke. Among the deficient mechanisms reported in hemiparesis, a reduction in presynaptic inhibition of group Ia terminals [17] and a decrease of post-activation depression [49], an increase of group I and II intersegmental excitatory influences [20,21] and changes in recurrent inhibition [50] could potentially increase the heteronymous facilitation and decrease the later inhibition.

### Correlations between coactivations and heteronymous modulation

Increased coactivation of plantarflexors during the knee extension task was correlated with the enhanced heteronymous facilitation in the paretic leg. This suggests that changes of transmission in intersegmental pathways linking quadriceps to soleus could participate in the abnormal coactivation of ankle extensors when knee extensors are voluntarily activated. One can hypothesize that, in severely affected stroke patients, contractions of knee extensors at high levels produce an overall facilitative intersegmental influence on plantarflexors via short propriospinal pathways, leading to their abnormal coactivation. Conversely, no correlation was found between the increased coactivation of knee extensors during the plantarflexion task and the heteronymous modulation that we studied. This absence of correlation during plantarflexion strengthens the hypothesis that the intersegmental pathway that we stimulated is a propriospinal pathway specifically linking quadriceps afferents and soleus motoneurones. This also suggests that the increased coactivation of knee extensors during plantarflexion may involve other spinal pathways than those tested in the present study. These other pathways may modulate the activity of knee extensors by transmitting the influences from ankle extensors. Such intersegmental pathways do exist in humans, in whom quadriceps motoneurones receive both excitatory and inhibitory influences of group Ia afferents and recurrent inhibition from soleus, respectively [18,51].

### Relations between spinal changes and motor deficits

This is the first time, to our knowledge, that changes in intersegmental pathways regulating the activity of motoneurones of muscles acting at different joints have been related to the impairment of selective muscular activation after stroke. An abnormal increase in the intersegmental facilitation of quadriceps activity by group I and group II afferents from common peroneal nerve was found at rest and during gait in stroke patients, but was not correlated to motor deficits [20-22]. Some evidence suggests that intersegmental pathways may have a relevant functional role. These pathways, which are modulated during voluntary contractions [52,53] and according to postural tasks [37], are thought to assist bipedal stance and gait [18,37,51]. Intersegmental pathways are under the regulation of descending and peripheral influences [54]. Changes in supraspinal influences consecutive to stroke may affect the regulation of these pathways [55] and contribute to the establishment of abnormal muscle activation patterns described in hemiparesis [56].

### Functional considerations

Coactivation of plantarflexors during the knee extension task was correlated with self-selected gait speed in stroke individuals. The higher was the coactivation, the slower the gait speed. This is a new finding; it suggests that the inappropriate coactivation of leg extensors, as revealed in static conditions in the present study, may impede the accomplishment of a dynamic task such as gait. Knee and ankle extensors are both anti-gravity muscles that have a usual out-of-phase reciprocal activation during gait with quadriceps and calf muscles reaching their peak activation at the beginning and at the end of the stance phase, respectively [5,6]. Premature activation of ankle extensors as the limb is loaded is the expression of an abnormal coactivation of these muscles at the early stance phase in the paretic side of stroke patients [3,4]. This coactivation has often been reported in the paretic leg [57,58], and is thought to be a major component of gait disorders after stroke [7]. Our results suggest that the malfunction of intersegmental influences of quadriceps afferents projecting to soleus motoneurones may participate in this premature activation of ankle extensors during peak activation of quadriceps at the early stance phase of hemiparetic gait.

Another finding of this study was the lower coactivation of biarticular muscles compared to the coactivation of monoarticular muscles in both sitting tasks on the Biodex, in healthy and stroke participants. This may reveal a differential control over mono- and biarticular muscles in this particular type of motor task, which requires torques to be produced in a specific direction at a single joint. Descending controls may favor the inhibition of biarticular muscles in these specific tasks since their activation would produce unwanted torques in other joints and thus reduce biomechanical efficacy.

Furthermore, clinical measures were more correlated with the levels of coactivation of biarticular muscles than those of monoarticular muscles, in both motor tasks assessed. The lower was the motor function, the higher the level of coactivation of biarticular leg muscles in stroke participants. This suggests the importance of the control of biarticular muscles in leg function. Impairment of the specific control of biarticular muscles after stroke could contribute to motor deficits in hemiparesis, particularly in coordination of the lower limb. Several motor control studies have underlined the specific role of biarticular muscles in force orientation and energy distribution across joints in the lower limb [59,60]. This control may be impaired in hemiparesis as suggested by an alteration of the normal phasic modulation of the activity of biarticular muscles at the paretic leg during a pedaling task [61].

Only changes in the intersegmental modulation from quadriceps to soleus (a monoarticular muscle) have been explored in the present study. However, changes in intersegmental modulation of biarticular muscles in the paretic leg have not been investigated. When considering the strong relationship between the coactivation of biarticular muscles and motor function found in our study, changes in spinal modulation of these muscles might be strongly related with motor function of the paretic leg. Such modulation involving spinal pathways liking quadriceps to gastrocnemius does exist in humans [18,51]. Future studies should particularly explore whether changes in pathways projecting from and to biarticular muscles and from distal to proximal limb segment are related with motor function of the paretic leg.

## Conclusion

There is an abnormal coactivation of knee and ankle extensors during maximal static contractions of the paretic leg of stroke patients with coordination deficits. Alterations of intersegmental pathways linking quadriceps to soleus are specifically correlated with the increased coactivation of ankle extensors during the knee extension task. Thus, these propriospinal pathways may participate in abnormal coactivations of ankle extensors while knee extensors are voluntarily activated in the paretic leg. Coactivations of biarticular muscles during static tasks directed at a single joint appear to be more related to motor deficits after stroke, and thus may have more functional impacts than coactivations of monoarticular muscles. Future studies should investigate the role of these and other intersegmental pathways in the motor deficits observed during dynamic conditions such as gait after stroke and in other central nervous system lesions.

## Declaration of competing interests

The authors declare that they have no competing interests.

## Authors' contributions

JOD prepared subjects, carried out the experiments, collected and analyzed the data, and drafted the manuscript. EM prepared subjects, carried out the experiments and collected the data. SAM analyzed the data and helped draft the manuscript. DB designed the experiments and helped draft the manuscript. RF designed the experiments, analyzed the data and helped draft the manuscript. All authors read, edited, and approved the final manuscript.
